# CT-scan *vs*. 3D surface scanning of a skull: first considerations regarding reproducibility issues

**DOI:** 10.1080/20961790.2017.1334353

**Published:** 2017-06-13

**Authors:** Stella Fahrni, Lorenzo Campana, Alejandro Dominguez, Tanya Uldin, Fabrice Dedouit, Olivier Delémont, Silke Grabherr

**Affiliations:** a School of Criminal Justice, University of Lausanne, Lausanne-Dorigny, Switzerland; b Department of Forensic Imaging, University Centre for Legal Medicine Lausanne-Geneva, Lausanne, Switzerland; c Department of Technological Radiology, Health School of Sciences Vaud - HESAV, Lausanne, Switzerland

**Keywords:** Forensic imaging, multi-detector computed tomography (MDCT), 3D surface scanning, anthropology

## Abstract

Three-dimensional surface scanning (3DSS) and multi-detector computed tomography (MDCT) are two techniques that are used in legal medicine for digitalizing objects, a body or body parts such as bones. While these techniques are more and more commonly employed, surprisingly little information is known about the quality rendering of digitalized three-dimensional (3D) models provided by each of them. This paper presents findings related to the measurement precision of 3D models obtained through observation of a study case, where a fractured skull reconstructed by an anthropologist was digitalized using both post-mortem imaging methods. Computed tomography (CT) scans were performed using an 8-row MDCT unit with two different slice thicknesses. The variability of 3D CT models superimposition allowed to assess the reproducibility and robustness of this digitalization technique. Furthermore, two 3D surface scans were done using a professional high resolution 3D digitizer. The comparison of 3D CT-scans with 3D surface scans by superimposition demonstrated several regions with significant differences in topology (average difference between +1.45 and −1.22 mm). When comparing the reproducibility between these two digitalizing techniques, it appeared that MDCT 3D models led in general to greater variability for measurement precision between scanned surfaces. Also, the reproducibility was better achieved with the 3D surface digitizer, showing 3D models with fewer and less pronounced differences (from +0.32 to −0.31 mm). These experiments suggest that MDCT provides less reproducible body models than 3D surface scanning. But further studies must be undertaken in order to corroborate this first impression, and possibly explain the reason for these findings.

## Introduction

Forensic imaging tends to be more and more used in legal medicine and forensic sciences [1–6]. Many different techniques exist and some of them are currently available and routinely employed in different forensic centres [1–4,6].

The most commonly used technique is post-mortem computed tomography (PMCT) [4,7]. This technique provides a rapid documentation of a body, allowing to store it digitally and to view the interior of it [3]. Although it is not the preferred method used for viewing internal organs  [8], it gives a first glimpse at major malformations, presence of blood or bone fractures etc. [9–11]. Additionally, it allows performing three-dimensional (3D) reconstruction showing the surface of the scanned body as well as the internal organs [3,12,13]. This possibility to represent the body, and especially the skeletal system in 3D, is an advantage often used for example to show fracture systems [14], ballistic trauma or to perform so-called “virtual anthropology” by investigating the virtual skeletons obtained using 3D volume rendering methods [15–23].

Another method of 3D imaging is three-dimensional surface scanning (3DSS). This technique was adapted for use from the industry  [24] and is employed today for medico-legal purposes especially in Switzerland [25–29]. The three main fields of application of this technique are traffic accident reconstructions  [25,26], correlation between a wound and an injury causing instrument  [28], and the comparison of bite marks with the suspect's dentition  [27]. It has also been employed by anthropologists and anatomists to digitally document bones  [30]. Concerning 3DSS, different technical systems exist. Today, the most commonly used methods for scanning are fringe-pattern technology  [26] or other patterned light systems such as mobile hand scanners  [31].

Although much literature exists on different techniques about how to obtain “virtual skeletons”, to our knowledge there is no information available on the quality of digitized 3D models in terms of repeatability, reproducibility and robustness of the digitalization process [30,32]. The objective of this technical note is to provide a first overview on data comparison between digital 3D models obtained by MDCT-scanning and 3DSS. Thus, we will discuss the influence of technical parameters on the resulting models looking at experience-based specific cases.

## Material and methods

MDCT is routinely performed on all human bodies admitted for medico-legal examination at our centre of expertise. This technique is also employed on the bones examined by the anthropologist  [15,30]. The present case study analysis and discussion was performed on a skull after maceration. The victim suffered blunt force trauma located on the left frontal bone, which was most likely to be the individual's cause of death. The skull was reconstructed including all fragments by a board-certified anthropologist using white glue. All the following investigations were performed once these procedures were done.

### MDCT-scanning

Three MDCT-scans were performed by a board-certified radiographer using an 8-row MDCT unit (LightSpeed, General Electric Healthcare). The three scans were performed with an interval of 48 days between the first and the second one, and only a day between the second and the third one. The acquisition protocols used differed from one scan to another, especially concerning the slice thickness. It varied from 1.25 mm (Scan 1) to 0.625 mm (Scan 3). The detailed acquisition parameters can be found in Table 1.

**Table 1.** Scan parameters of CT-scans 1-3 (Ge HealthCare LightSpeed - 8 rows).

CT-scan	Scan type	Thickness slice Table speed Pitch	Interval spacing	Scan Field of view (FOV)	Kilo Volts (kV)	Milli Amperage (mA)	Algorithm of reconstruction	Position of acquisition
CT-scan 1 Date 11.09.2014	Helical 1.0 s	1.25 mm 6.25 0.625:1	0.6	Head 25	140	140	Bone +	Axial
CT-scan 2 Date 29.10.2014 (+ 48 days)	Helical 1.0 s	1.25 mm 6.25 0.625:1	0.6	Head 25	140	140	Bone +	Coronal
CT-scan 3 Date 30.10.2014 (+ 49 days)	Helical 1.0 s	0.625 mm 1.25 1:1	0.3	Head 25	140	140	Bone +	Coronal

**Figure 1.**
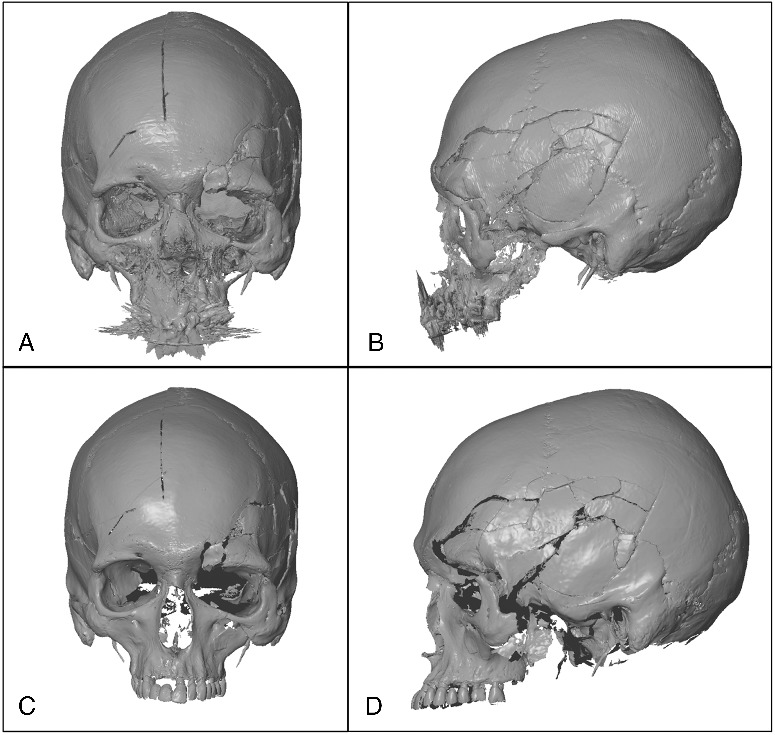
3D Model obtained with the CT-scan 1: (A) front view; (B) profile view. 3D Model obtained with the 3D surface scanner 1: (C) front view; (D) profile view.

In order to generate 3D models for computed tomography (CT) raw data, we used the OsiriX MD imaging software (Free version 5.6). Volume rendering was done followed by surface rendering (high resolution and pixel value 200; Figure 1(A,B)). The file was then exported to STL format (STereoLithography, Standard Tessellation Language) in order to compare CT-data with 3D surface scan data.

### 3DSS

For the 3D surface scanning, a GOM ATOS Compact Scan 5M was employed by a trained operator. Measuring Volume of 300 (300 mm × 230 mm × 230 mm) and 1.5 mm reference point (white diameter) were chosen in order to obtain a reachable resolution of 0.124 mm. The two different 3D surface scans were performed with the same parameters, the same instrumentation and by the same trained operator. The first 3DSS was performed on 19 September 2014 (Figure 1(C,D)) and the second 40 days later on 29 October 2014.

### Comparison between 3D models

Superimposition and comparison between both 3D models were done with a GOM ATOS software 7.5. A pre-alignment was performed using three identical points, and then a “Best fit alignment” was used over the surface as to obtain the best possible superimposition. All comparisons were made by the same experienced operator.

When using the surface comparison function of the GOM ATOS software 7.5, colours are mapped on the models. They provide an indication of the spatial correlation of the models, and allow a quick and efficient visual appreciation of the qualitative colour matching of the models. Regions, in which the surface is displayed in green colour, indicate almost no deviation between the two models. The red colour shows that there is a positive deviation as the borders of the first model are overlapping those of the second one. Blue colour indicates a negative deviation as the borders of the first model are not reaching the borders of the second one. A punctual value for the deviation can be highlighted by clicking on an area.

## Results and discussion

If we only focus on the single aspect of 3D modelling, all of them seem to represent the object in details. The only visualization problem that could appear would be the 3D rendering of MDCT, when inlay dental material creates some types of artefacts on the model that do not allow the exact representation of the tooth's shape. These hardening artefacts are due to the interaction of X-rays with some metallic component of the treated tooth  [33].

To evaluate the precision of the obtained images, it is important to compare on one hand scans performed at different time lags with the same technique, and on the other hand to compare the results obtained with the different methods. A thorough examination of the accuracy would definitely require to perform a set of scans for each of the considered techniques. In this regard, our study does not claim to provide such a complete assessment, but it shed lights on possible issues that would require further clarifications.

### Comparison between MDCT models obtained at different time lags

**Figure 2.**
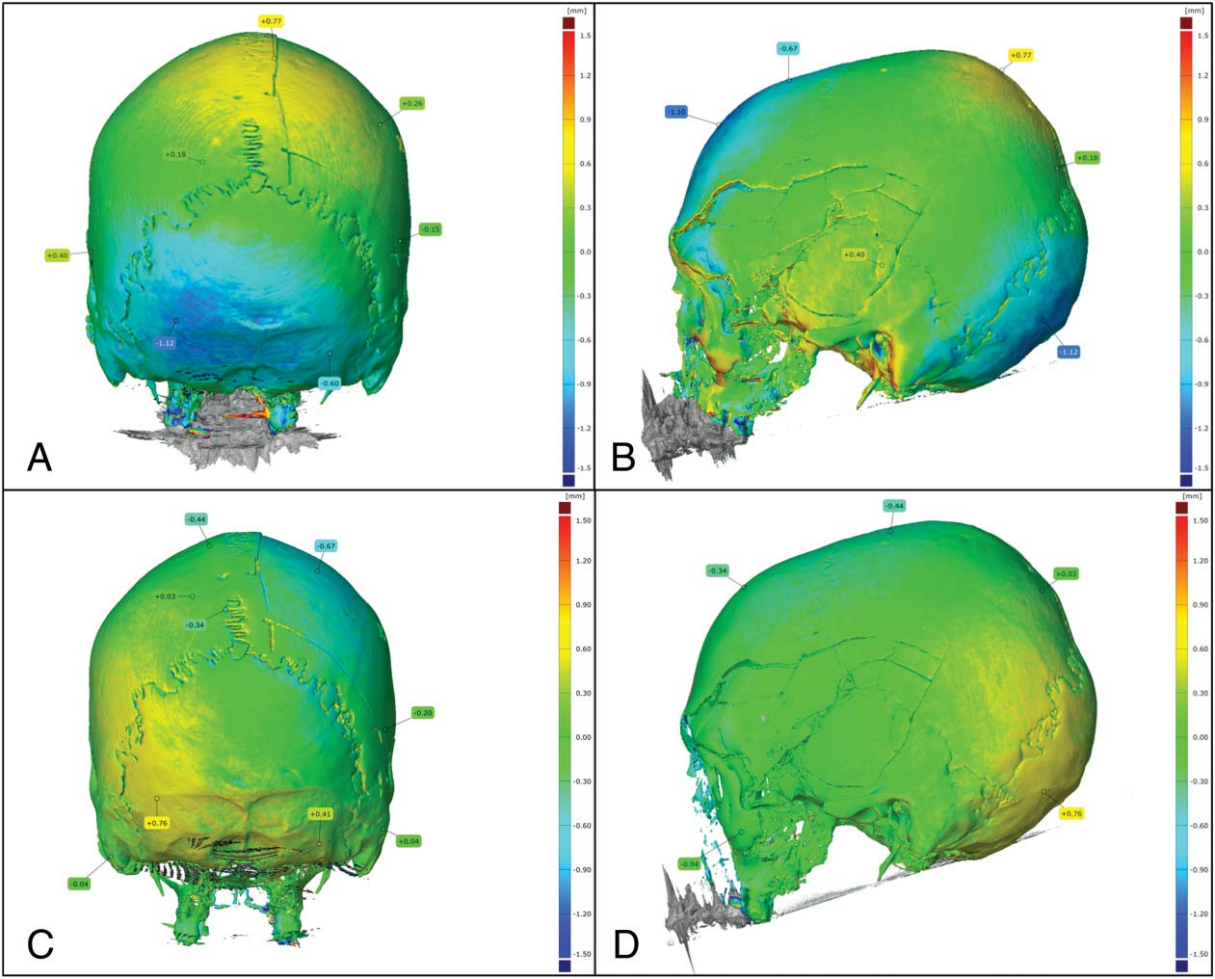
Comparison between the first and the second CT-scan (Δ*T* = 48 days): (A) posterior view; (B) profile view. Comparison between the second and the third CT-scan (Δ*T* = 1 day): (C) posterior view; (D) profile view.

This comparison shows generally a great difference in some areas of the 3D model going from +1.58 to −1.64 mm in average. For example, in the occipital region, difference of −1.12 mm can be observed (Figure 2(A,B)). The comparison between model of the second and the third CT scan shows slightly better results with intervals of values from +0.76 to −0.67 mm (Figure 2(C,D)). This may be due to a thinner slice thickness of the third scan. In fact, as Dalrymple and collaborators explained in their article, the thinner the slice, the better the spatial resolution  [34]. This means that employing a slice thickness of 0.625 mm improves precision and reduces differences observed between MDCT-scans.

Although significant differences are observed between 3D models from MDCT-scans, they are still better than values obtained for dry bones in other research. In some studies like the one of Stull and collaborators  [35], authors demonstrated high accuracy between measurements taken from a dry element and measurements taken from the 3D-MDCT image of the same dry element. They compared morphological measurements performed manually from dry-bones with those performed by measuring the same parameters on 3D models obtained by MDCT-scan of the same bones. They found that measurement differences were in acceptable range for anthropologist (<2 mm). Therefore, they assessed that CT images are accurate representations of the true objects’ dimensions  [35]. They also claim that differences were rather resulting from human error than from imaging technology. However, they did not compare MDCT-scan of the same dry bone. In our case, the variations observed were too high for the same bone measured. And as the measurements are calculated by the software, this eliminates the human error from the hypothesis. We do not think that such differences between MDCT-scans are coming from the scanning step but rather from the volume rendering one. Thus, one hypothesis would be that differences would result from the OsiriX MD software when creating the 3D model algorithms. In fact, it can be deduced that OsiriX MD is creating information from MDCT-scan data without using the information from the real bone. Furthermore, artefacts like holes, absence or exaggeration of details can also be produced during this process  [36]. Another hypothesis would be that encountered difficulties could be partially due to the reconstruction of the skull using the glue which could have led to changes when hardening.

### Comparison between 3DSS models obtained at different time lags

**Figure 3.**
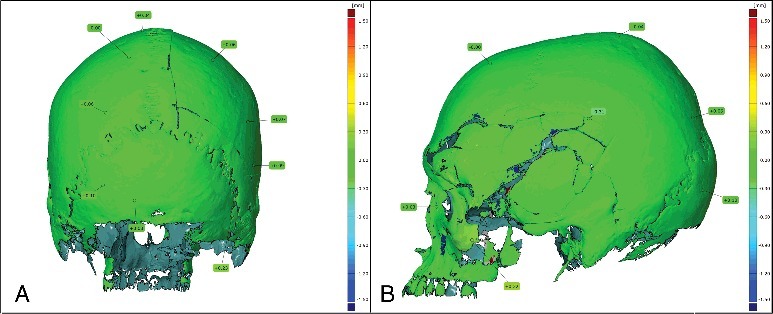
Comparison between the first and the second 3D surface scan (Δ*T* = 40 days): (A) posterior view; (B) profile view.

This comparison shows only small differences on rare parts of the surface. Indeed, most of the superimposition is green and the values are going from +0.32 to −0.31 mm (Figure 3). These observations can be explained by the fact that 3D models from 3D surface scans are directly obtained while scanning; unlike MDCT-scans, where volume rendering is done from MDCT images using some complex algorithm. In fact, the GOM ATOS Compact Scan creates the 3D model directly using the triangulation principle. Therefore, it can be viewed on the screen even during the scanning process.

### Comparison between MDCT and 3DSS models

**Figure 4.**
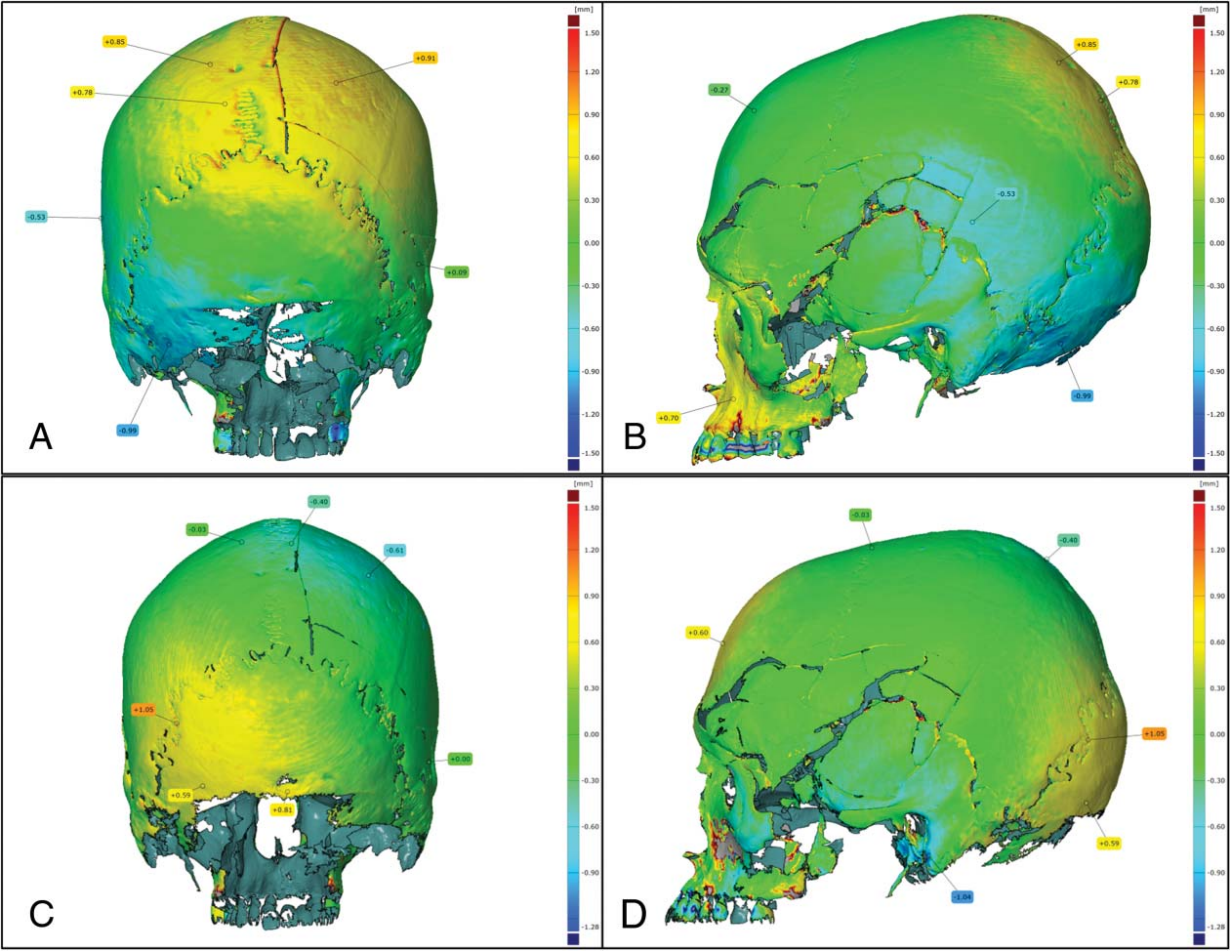
Comparison between the first CT-scan and the first 3D surface scan: (A) posterior view; (B) profile view. Comparison between the second CT-scan and the second 3D surface scan: (C) posterior view; (D) profile view.

This superimposition allows detecting an average difference between +1.45 and −1.22 mm in several areas. For example, a difference of −0.99 mm is observed in the occipital region (Figure 4(A,B)). When superimposing the 3D models of second MDCT-scan and the second 3D surface scan, still high differences are observed: the interval is going from +1.05 to −1.04 mm in average (Figure 4(C,D)).

The 3D models of the investigated skull showed clear differences if performed by MDCT or 3D surface scanning. It is now important to understand which parameters have an influence on them. While the first CT and 3D scan were not implemented on the same table nor in the same position, the second CT and 3D scan were done on the same conditions, and performed directly one after the other. Even then, differences were observed between the obtained 3D models, although they were less important.

Similar differences were observed between different MDCT-scans while no significant differences were seen between different 3D surface scans. This suggests that the repeatability and reproducibility of 3DSS is higher than the MDCT one. However, it can also be argued that the use of a thinner slice thickness for MDCT-scans could provide improvement not only on the spatial resolution but also on the quality of the resulting 3D model, getting it closer to the results obtained by 3DSS.

Differences between the two techniques could also be explained by their functioning. If we look at the 3D surface scanner, 3D models are directly created while scanning, but for the CT Scanner, 3D models are obtained indirectly from calculation after scanning. CT Scanner produces slice models that are then assembled together.

The superimposition of the models using GOM ATOS software 7.5 allowed us to be attentive to such differences and led us to identify this comparative case study.

### Limitations

One of the major limitations of this study is the fact that all observations were made on one single case. However, these first observations gave us new interesting results that, in our opinion, should be further investigated.

## Conclusion

Our research study, despite the limitations previously exposed, lead us to the hypothesis that models generated by the combination of 8-row MDCT and OsiriX MD data treatment have a lower reproducibility than 3D surface scanning. This suggests that 3DSS is more suitable than MDCT-scanning to obtain detailed digital 3D models for anthropological investigations of bones

This first impression would require further studies on a more extensive set of samples, taking into account different acquisition parameters.
